# The NFκB Antagonist CDGSH Iron-Sulfur Domain 2 Is a Promising Target for the Treatment of Neurodegenerative Diseases

**DOI:** 10.3390/ijms22020934

**Published:** 2021-01-19

**Authors:** Woon-Man Kung, Muh-Shi Lin

**Affiliations:** 1 Department of Exercise and Health Promotion, College of Kinesiology and Health, Chinese Culture University, Taipei 11114, Taiwan; nskungwm@yahoo.com.tw; 2 Division of Neurosurgery, Department of Surgery, Kuang Tien General Hospital, Taichung 43303, Taiwan; 3 Department of Biotechnology and Animal Science, College of Bioresources, National Ilan University, Yilan 26047, Taiwan; 4 Department of Biotechnology, College of Medical and Health Care, Hung Kuang University, Taichung 43302, Taiwan; 5 Department of Health Business Administration, College of Medical and Health Care, Hung Kuang University, Taichung 43302, Taiwan

**Keywords:** neurodegenerative disease, CISD2, proinflammatory response, mitochondrial dysfunction, NFκB

## Abstract

Proinflammatory response and mitochondrial dysfunction are related to the pathogenesis of neurodegenerative diseases (NDs). Nuclear factor κB (NFκB) activation has been shown to exaggerate proinflammation and mitochondrial dysfunction, which underlies NDs. CDGSH iron-sulfur domain 2 (CISD2) has been shown to be associated with peroxisome proliferator-activated receptor-β (PPAR-β) to compete for NFκB and antagonize the two aforementioned NFκB-provoked pathogeneses. Therefore, CISD2-based strategies hold promise in the treatment of NDs. CISD2 protein belongs to the human NEET protein family and is encoded by the *CISD2* gene (located at 4q24 in humans). In CISD2, the [2Fe-2S] cluster, through coordinates of 3-cysteine-1-histidine on the CDGSH domain, acts as a homeostasis regulator under environmental stress through the transfer of electrons or iron-sulfur clusters. Here, we have summarized the features of CISD2 in genetics and clinics, briefly outlined the role of CISD2 as a key physiological regulator, and presented modalities to increase CISD2 activity, including biomedical engineering or pharmacological management. Strategies to increase CISD2 activity can be beneficial for the prevention of inflammation and mitochondrial dysfunction, and thus, they can be applied in the management of NDs.

## 1. Preface

In the central nervous system (CNS), various complex neurodegenerative processes, including chronic inflammation, continuously occur with natural aging [1].

However, in neurodegenerative diseases (NDs), these processes are much more rapid and pronounced. NDs combined with cognitive or motor deficits are disastrous for patients and contribute a significant burden to the entire society.

Proinflammatory response and mitochondrial dysfunction are driven by activation of nuclear factor kappa-light-chain-enhancer of activated B cells (NFκB) and have been linked to NDs [2,3,4]. Any novel strategy to suppress NFκB signaling and prevent inflammation and mitochondrial dysfunction may have a therapeutic effect on NDs. This review has focused on CDGSH iron-sulfur domain 2 (CISD2) as a unique zinc finger and iron-sulfur protein and emphasized its protective role against NFκB activation. The positive findings regarding the protective role of CISD2 against inflammation and mitochondrial dysfunction support the idea that CISD2 can be a therapeutic target for NDs.

## 2. Inflammation and Mitochondrial Dysfunction Implicated in NDs

In the pathogenesis of NDs, various microneural abnormalities are involved in structural and functional impairment or loss of CNS neural networks such as enhanced permeability of the blood-brain barrier, damaged axons or myelin sheaths, dysregulated immune activation (innate and adaptive), and neuronal insults [5,6]. The abovementioned pathological features commonly underlie NDs such as Alzheimer’s disease (AD), Parkinson’s disease (PD), multiple sclerosis (MS), and amyotrophic lateral sclerosis (ALS).

Miscellaneous factors, such as genetics, aging, or acquired environmental factors, cause NDs [7]. The well-documented genetic manifestations include the following: (1) autosomal dominant spinocerebellar ataxia type 1 (SCA1): *ATXN1* mutation (associated with polyglutamine repeat in ataxin-1) [8]; (2) autosomal dominant early-onset AD: mutations in amyloid precursor protein (chromosome 21), presenilin-1 (chromosome 14), and presenilin-2 (chromosome 1) [9]; (3) autosomal dominant early-onset PD: point mutations in *SNCA* encoding α-synuclein [10]; (4) autosomal recessive early-onset PD: mutation in *PARK2*, *PINK1*, and *PARK7* (encoding parkin, PINK1, and DJ-1 respectively) [11]; early-onset familial forms of ALS: mutation in *superoxide dismutase 1* (*SOD1*) [12]; and (5) autosomal dominant frontotemporal dementia: mutations in the progranulin (*PGRN*) gene (chromosome 17q21), microtubule-associated protein tau (*MAPT*) P301L (chromosome 17q21), and chromosome 9 open reading frame 72 (*C9orf72*) (chromosome 9p21) [13].

The aging process in the CNS has been indicated to precede NDs and is a significant risk factor for NDs. During brain aging, the CNS-resident innate immune cells microglia switch toward detrimental M1 and diminish beneficial M2 phenotypes. Dysfunctional microglia exhibiting resistance to immune adaptive regulation and excessive immunocompetence are highly related to the upregulation of inflammatory genes and, thus, cause persistent neuroinflammation in the CNS [14,15]. Environmental and particularly genetic factors can result in abnormal conformational changes in certain proteins, leading to intracellular and extracellular protein aggregation, which promote neurodegeneration [16]. The abnormally folded/aggregated proteins underlying NDs include hyperphosphorylated tau, Aβ-peptide (for AD), α-synuclein (for PD), huntingtin (for Huntington disease), and prion (for prion diseases) [17]. Based on this immunocompetent response, aggregation of pattern recognition receptors (PRRs), which are mainly toll-like receptors (TLRs), activate microglia and consequently trigger neuroinflammation [18]. Stimulated M1 phase microglia have been indicated to activate astrocytes directly [19]. Glia-mediated neuroinflammation has been linked to the pathogenesis of various NDs, including AD, PD, and MS [20,21]. These pathological mechanisms, which are consequently detrimental to neuronal survival, involve the generation of various toxic substances, such as reactive oxygen species (ROS), nitrogen species, and proinflammatory mediators [22]. During neuroinflammation, microglia-astrocyte crosstalk facilitates reciprocal modulation of the innate immune defense in the CNS. Stimulated microglia facilitate subsequent astrocytic activation and manipulate the role of astrocytes in neuroprotection or neurotoxicity [23]. Through autocrine feedback, astrocytes inhibit astrocytes [24] or microglial activities and functions [25].

Prolonged neuroinflammation has been demonstrated to result in mitochondrial dysfunction, including altered mitochondrial dynamics, enhanced mitochondrial membrane permeabilization, oxidative phosphorylation, and ROS production [2,26]. As a kind of damage-associated molecular pattern (DAMP), contents of injured mitochondria, such as mitochondrial debris, mitochondrial DNA, and cardiolipin, which are removed by mitophagy, further promote the activation of NACHT, LRR, and PYD domains containing protein 3 (NLRP3) inflammasomes [27]. Consequently, pro-caspase-1 activation and subsequent production of proinflammatory cytokines (IL-1β, IL-18) combined with microglial TLR-9 lead to the profound release of proinflammatory mediators [28]. This vicious cascade may expand with the release of more mitochondrial DAMPs, more enhanced inflammasome activation, and more extensive glial neuroinflammation [29]. In summary, pathological mechanisms underlying NDs involve inflammatory responses [30,31] and mitochondrial dysfunction [32]. Hence, treatment strategies that exhibit anti-inflammatory and protect mitochondrial function could be potentially beneficial for the management of NDs.

## 3. NFκB-Driven Inflammation and Mitochondrial Dysfunction in NDs

The two critical pathogeneses involved in NDs, inflammation and mitochondrial dysfunction, are provoked by NFκB. The ND-NFκB axis provides strong rationale for further investigation to modulate NFκB as a therapeutic target in NDs. NFκB serves as a transcription factor in organisms. By combining with a specific nucleotide sequence upstream of the genetic code, NFκB can regulate the transcription of genes involved in various vital functions such as inflammation, innate and adaptive immunity, cell differentiation, proliferation, and apoptosis [33].

Structurally, NFκB is a kind of heterodimer or homodimer composed of the following five different subunits: c-Rel, Rel-A (same surname p65), Rel-B, NFκB 1 (same surname p50), and NFκB 2 (same surname p52) [34]. Specifically, p50 and p52 are derived from proteolytic processes of the large precursor proteins p105 and p100, respectively. Each NFκB subunit has been shown to contain the Rel homology domain (RHD) at the N-terminal region [4]. The following functions have been linked to the sequences of RHD: (1) DNA binding; (2) dimerization; (3) binding of inhibitors (IκB proteins) to deactivate nuclear localization signal (NLS) in unstimulated status [35]; and (4) NLS, which guides NFκB translocation from cytoplasm to the nucleus in stimulated status.

The three subunits, c-Rel, p65 (Rel-A), and Rel-B, are characteristic of a transactivation domain (TAD) [36], which serves as an activation zone through the combination with various kinds of transcriptional factors, such as transcriptional co-activators cAMP-response-element-binding-protein-binding protein (CBP). Through the connection of TADs with transcriptional factors, transcription reactions can occur.

As the well-documented pathway of canonical NFκB activation, various PRRs act as environmental stimuli to immune cells (such as pathogen-associated molecular patterns, or DAMPs) to activate the IκB kinase (IKK) complex. IKK-associated cytoplasmic signals result in IκB (an inhibitor of κB) phosphorylation and subsequent degradative ubiquitination. As described previously, IκB degradation leads to unmasking of NLPs in p65/p50 or p50/c-Rel dimers, nuclear translocation of NFκB from cytoplasm, and consequent proinflammatory signal transduction [33]. Specifically, the IKK complex consists of the following three subunits: IKK-α, IKK-β, and IKK-γ. IKK-α and IKK-β serve as catalytic components of the IKK complex. In addition, IKK-γ has been shown to contain a C-terminal zinc finger domain of the C2HC type [37]. The zinc finger of IKK-γ potentially upregulates or downregulates the activity of IKK complexes by binding to upstream proteins. Ubiquitin recognition and the binding of IKK-γ zinc fingers are related to the regulation of NFκB, and thus, IKK-γ is considered a NFκB essential modulator (NEMO).

In addition to the cytoplasm, NFκB is detectable in mitochondria [4]. It regulates mitochondrial function [3] and modulates the activities of NLRP3 inflammasomes in the endoplasmic reticulum (ER) and mitochondria-associated ER membranes (MAMs) [38]. In mitochondria, NFκB has been verified to be involved in the regulation of the following mitochondrial functions: mitochondrial dynamics, activities of respiratory complexes for electron transport chain, release of cytochrome C, and cellular apoptosis. Furthermore, an activated form of NLRP3 inflammasomes has been presented to migrate to the MAM from the ER membrane [38] after receiving priming signals, such as ligands of TLRs, mitochondrial ROS, and cytokines. Moreover, after activation of NLRP3 inflammasomes, advanced cytokine loops (IL-1β, IL-18) are subsequently formed, which can then stimulate the generation of proinflammatory cascades and eventually establish a vicious cycle between inflammation and mitochondrial dysfunction. NFκB-regulated NLRP3 inflammasomes activation involves the following critical organelles significant to cellular function: mitochondria, ER membranes, and MAMs [39].

## 4. The Evolutionary Conserved NEET Family Member CISD2 Regulates Important Homeostatic CNS Functions such as pH and Oxidation State

Cytogenetically, *CISD2* is located at the long arm of chromosome 4 at position 24 (4q24) in humans [40]. The synonyms of *CISD2* documented in the literature are as follows: nutrient-deprivation autophagy factor-1 (NAF-1), ER intermembrane small protein (Eris), mitoNEET related 1 (Miner 1), Wolfram syndrome 2 (WFS2), zinc finger, CDGSH-type domain 2 (ZCD2), and nervous system overexpressed protein 70 (Noxp70). Three small exons with an average size of 24 kb are included in the gene transcript of *CISD2.* Transcript accession identifiers of *CISD2* are as follows: NM_001008388.4 (NCBI databases), ENST00000273986, ENST00000503643, and ENST00000574446 (Ensembl databases) [41].

*CISD2* encodes the CISD2 protein, which is the second member of the three members of the human NEET family (CISD1-3); hence, it is called CISD2. In the NEET protein family, the following amino acid sequence commonly appears in the C-terminal of each family member Asn-Glu-Glu-Thr (NEET) [42]. Hence, the protein family is named NEET. Moreover, all the members of the NEET protein family share the following feature: (1) the sequence motif, CDGSH (39 amino acid stretch) [43]; (2) the combination of CDGSH domain with the [2Fe-2S] cluster through coordinates of 3-cysteine (Cys)-1-histidine (His) on the CDGSH motif [44]. Thus, the NEET protein family is characterized by the unique combination of 3Cys-1His CDGSH 2Fe-2S, [C-X-C-X2-(S/T)-X3-P-X-C-D-G-(S/A/T)-H] [45,46]. The CDGSH domain can be detected in archaea, bacteria, plants, and humans, which provides evidence of high evolutionary conservation [47].

In humans, three members of the NEET protein family, CISD1-3 [48,49,50], have been well documented. Due to the specific feature (CDGSH iron-sulfur domain), the three human NEET proteins are named CISD1-3 (based on the first letter of each word). The morphological differences of the NEET protein family can be divided into the following two categories [46]: (1) homodimer with each monomer with one CDGSH domain (Class I NEET proteins, e.g., CISD1 and CISD2); and (2) monomeric proteins with two CDGSH domains (Class II NEET proteins, e.g., bacterial mitochondrial inner NEET protein [MiNT] and CISD3).

On the coordination of (Cys)3(His)1-[2Fe-2S] cluster, the histidine ligand (His114) of CISD2 protein combines with the [2Fe-2S] cluster via Nδ [51,52]. Significantly, His114 influences the [2Fe-2S] cluster binding affinity according to the redox state of the cluster or environmental pH value. As the pH declines to 7, protonation of the histidine ligand can be detected in CISD2 proteins, which leads to the transfer of [2Fe-2S] cluster, i.e., cluster transfer [53]. The cluster transfer of NEET proteins is accelerated by reducing the pH levels [54].

In addition to the pH effect, the extent of the oxidation state of the [2Fe-2S] cluster influences NEET-cluster lability. The [2Fe-2S] cluster of NEET proteins has been shown to be structurally stable in the reduced state [55]. Under environmental stress, vital iron-sulfur proteins may be enzymatically inactivated, and the function is impaired due to the loss of [Fe-S] clusters caused by damage; for example, an apo-protein receptor involving in iron homeostasis, an inactive form of ion regulatory protein 1 (IRP1), and cytosolic aconitase [56]. The impaired cluster function of these damaged proteins can be rescued by NEET proteins as a potential reducing agent. The mitochondrial antioxidant effect of NEET proteins can be achieved by electron transfer from the cluster [57]. Specifically, the alternation in the redox status of [Fe-S] clusters contributes to cluster transfer [58,59,60]. [Fe-S] clusters change from a reduced (rested) to an oxidation (activated) state and subsequently result in cluster transfer to the apo-protein receptors. After the donation of clusters from NEET proteins, these injured proteins can potentially be repaired. With a protective effect commonly shared by the NEET family, the [2Fe-2S] cluster in CISD2 acts as a homeostasis maintainer under environmental stress through the transfer of electrons or iron-sulfur clusters.

Structurally, the CISD2 protein possesses a common frame with the NEET protein, folds into a homodimeric structure in a unique form, and generates the so-called NEET fold (two-fold pseudo symmetric backbone). The two specific domains, β-cap (comprising three long β-strands) and cluster binding (including C-terminal [2Fe-2S] CDGSH motif and α-helix structure), constitute the main structure of CISD2 in each monomer [47,53]. In general, a hydrophobic core has been detected in the β-cap domain. The cluster binding domain is hydrophilic and charged. These characteristics are related to protein folding and the combinations with specific molecules through the domains [61].

Most of the NEET conformation, including CISD, is situated in the cytoplasm, and these parts of the CISD2 protein form the cytosolic domain. In addition to these functional parts of NEET folds, CISD2 protein has transmembrane helices at their N-terminal region and in-organelle domain [47]. Each monomer of homodimeric CISD2 protein anchors to the outer membrane of mitochondria (OMM) through the transmembrane helix [53]. The other two subcellular locations reported in the literature include the ER and MAMs. Specifically, CISD2 has been proven to be an integral membrane protein with a C-terminal EGFP tag on ER membrane [50] and is abundant in MAMs [62,63].

The CDGSH motif of NEET fold in the CISD2 protein has been indicated to be a CDGSH-type zinc finger [45,64] with the characteristic of a CysCysCysHis finger [65] (serves as Cys3/His1 type, C3H1, among nine subgroups of all zinc finger proteins [66]). Although annotated as zinc fingers, the C3H1 ligand set coordinates with the [2Fe-2S] cluster instead of zinc in CISD2. It has been postulated that insults from oxidative stress or ROS formation may destroy the zinc finger structure, especially the coordinates of Cys and His [67]. Redox properties of [2Fe-2S] clusters is associated with the activity of zinc finger motifs of CISD2 [68].

## 5. Physiological Function of CISD2

In ER or MAMs, CISD2 mediates pivotal functions, including lipid synthesis and protein folding [64]. CISD2 can act as a transport conduit for redox calcium between the ER, MAMs, mitochondria, and the cytosol. As such, CISD2 can regulate redox reactions, which is critical for mitochondrial modulation relevant to NDs. [Fe-S] cluster transfer from CISD1 to CISD2 has been shown to achieve effective transport of labile iron from mitochondria to the cytosol to prevent mitochondrial Fe/ROS/Fe-S dyshomeostasis [69]. CISD2 mutation potential leads to mitochondrial iron overload and ROS toxicity [70]. Moreover, protection against apoptosis-related mitochondrial dysfunction of CISD2 in combination with BCL2 has been proven to be ineffective under the circumstances of CISD2 absence from the [2Fe-2S] cluster [71].

CISD2 is interesting for providing the following inhibitory functions to protect cells from damage:

(1) Calcium excitotoxicity. CISD2 has been shown to depress excitotoxic Ca^2+^ increase at the ER through the binding of CISD2 to BCL2 along with the inositol 1,4,5-triphosphate (IP3) receptor (a kind of calcium channel in the ER membrane) [72,73]. CISD2 deficiency can trigger an increase in ER and cytoplasmic Ca^2+^ levels in CISD2 knockout mice compared with wild-type mice [72]. Furthermore, results obtained from CISD2 knockout mice showed that CISD2 along with GTPase of immune-associated protein 5 (Gimap5) in MAMs decreased cytosolic Ca^2+^ surge and enhanced mitochondrial Ca^2+^ uptake [63].
(2) Apoptosis. CISD2 along with the IP3 receptor has been shown to connect with a combination of Bcl-2-Beclin-1 complex in the ER membrane. Bcl-2 exerts its antiapoptotic effect via antagonism of Beclin-1. As a Bcl-2-interacting protein, CISD2 functions as an autophagy regulator. The binding of CISD2 to Bcl-2 regulates Bcl-2 to antagonize autophagy/apoptosis in response to stress [71,74]. CISD2 enhances Bcl-2-Beclin-1 interaction and prevents the potential apoptotic cellular damage. When CISD2 is attenuated, the interaction between Bcl-2 and Beclin-1 is greatly reduced, and autophagy is triggered. As mentioned earlier, the effect generated by the combination of CISD2 and Bcl-2 is invalid when CISD2 loses the [2Fe-2S] cluster [71].
(3) OMM breakdown and subsequent mitochondrial abnormality. The most prominent study on mitochondrial CISD2 function from *CISD2* knockout mice was published by a group of Taiwanese neuroscientists headed by Ting-Fen Tsai in 2009 [75]. As a part of OMMs, CISD2 proteins play a vital role in the maintenance of mitochondrial function. Studies on *CISD2*^-/-^ mice have demonstrated that *CISD2* knockout induces mitochondrial degeneration, autophagy, and consequent enhancement of the aging process. As a kind of ER/mitochondria-related disease, WFS2, a subtype among WFS (featured in diabetes insipidus, diabetes, optic atrophy, and deafness [DIDMOAD]), has been linked to the recessive mutation of *CISD2*. WFS2 may clinically manifest as diabetes mellitus, optic atrophy, and a bleeding tendency [76].

## 6. CISD2 as a NFκB Antagonist against Inflammation and Mitochondrial Dysfunction: A Promising Target for NDs

The research team has addressed the vital role of CISD2 protein in preventing inflammation and mitochondrial dysfunction. A *CISD2* knockdown model using siCISD2 in immune and non-immune cells was applied to verify the potential function of CISD2. The following findings were revealed: (1) enhanced inflammatory responses (elevated production of iNOS and chemokine regulated on activation, normal T cell expressed and secreted [RANTES]), extensive mitochondrial dysfunction (including decreased mitochondrial membrane potential DeltaPsi(m) and elevated ROS release), promoted apoptosis, and decreased cell viability in neuron-like cells, SH-SY5Y [77,78]; and (2) augmented inflammatory reaction, as indicated in enhanced M1 microglia polarization (increased expression of TNF-α, IL-1β, iNOS, and COX2), attenuated M2 microglia phenotype (decreased expression of Arg-1, Ym1, and IL-10), and promoted apoptosis in EOC microglial cells [79].

Moreover, the anti-inflammatory effect of CISD2 has been shown to antagonize the activation of NFκB. Enhanced activity of NFκB p65 subunit DNA binding and subsequent nuclear translocation of NFκB p65 have been confirmed in siCISD2-transfected EOC microglial cells [79]. CISD2 inhibits NFκB signaling by acting upstream of the peroxisome proliferator-activated receptor (PPAR)-β (synonym: PPAR-δ), which is due to the decrease of PPAR-β expression shown in SH-SY5Y with *CISD2* knockdown [80]. The beneficial effect of PPAR-β has been demonstrated to prevent IκB degradation and consequent NFκB activation [81,82]. It has been established that the anti-inflammatory effect of CISD2 is through the upstream regulation of PPAR-β/NFκB signaling.

## 7. CISD2 Attenuation on Neural Pathology

As a regulator of NFκB activation, CISD2 expression levels have been demonstrated to decrease during neurological pathology. CISD2 attenuation leads to reduced suppression against NFκB activation. Therefore, the proinflammatory response and mitochondrial dysfunction caused by NFκB are manifested in the cytoplasm and mitochondria. It is worth noting that the inflammation and mitochondrial dysfunction caused by CISD2 decline are widely involved in neurodegenerative states and neurotrauma. Specifically, CISD2 expression was found to be decreased in the brain and spinal cord of aging mice compared to that in the brain and spinal cord of young mice. A reduction of CISD2 expression and enhanced production of proinflammatory mediators were confirmed in the group of 35 days in vitro (DIV) long-term primary culture of astrocyte compared to those in the group of 7 DIV cells [78]. Similar effects for age-dependent CISD2 decline have been reported in mouse tissues of the brain, skin, and skeletal muscles [83]. In addition, cell injured models of LPS challenge or animal models of spinal cord hemisection have been shown to reduce the expression levels of CISD2 in primary astrocytes [77], ALT astrocytes [80], and mice [77,80], respectively. Therefore, CISD2 decline can be observed in neural pathology involving either the neurodegenerative process or neurological trauma. As mentioned above, detailed pathological effects of inflammation and mitochondrial dysfunction caused by insult-attenuated CISD2 expression are well described in cell experiments with *CISD2* knockdown.

## 8. CISD2-Elevating Strategy as the Potential Future Therapy in NDs

NFκB-evoked critical mechanisms, inflammation and mitochondrial dysfunction, underlie the pathogenesis of NDs. The protective effect of CISD2 has been established to inhibit the above two pathological mechanisms and NFκB activation. Hence, CISD2 is considered as a potential therapeutic target for NDs. As discussed above, CISD2 expression is reduced under the circumstances of CNS degenerative status or injuries. Therapeutic strategies that focus on how to increase the expression level of CISD2 are promising for the future treatment of NDs. Below, we address the previously reported CISD2-elevating strategies.

**Strategy 1. Experimental Over-Expression**

When CISD2 is overexpressed in AD mice, reduced neuron loss, β-amyloid-induced mitochondrial dysfunction, and decreased immunofluorescence of Iba1 (ionized calcium-binding adapter molecule 1) and GFAP have been reported in the hippocampus [84].

**Strategy 2. Experimental Cryogen Spray Cooling**

Cryogen spray cooling has been used for the management of spinal cord injury, showing protection against injury-induced CISD2 decline and astrocyte-mediated neuroinflammation in a rat sustained spinal cord hemisection [85].

**Strategy 3. Curcumin**

Studies published in the literature support that treatment strategies of raising CISD2 have a protective effect on CNS injuries and diseases. Aged mice (104 weeks) and 35 DIV primary cultured astrocytes have shown to have decreased CISD2 expression levels; such aging-driven CISD2 loss can be attenuated after curcumin treatment in vivo and in vitro [78]. Similar effects have been seen in injury experimental models. Curcumin has been demonstrated to reduce CISD2 loss in mice with spinal cord injury and LPS-challenged astrocytes [77].

**Strategy 4. Momordica Charantia Linn. var. Abbreviata Ser. (WBM)**

A similar pattern of reduction in CISD2 protein after SCI and in LPS-stimulated astrocytic cell lines (ALT) also improves by the treatment of WBM. The decrease in glial activation and proinflammatory cascades is also accompanied by an increase in the levels of CISD2 protein after WBM treatment [80].

As mentioned above, CISD2 can be pathologically attenuated in either neurodegenerative or neurotraumatic status and cause NFκB-provoked inflammation and mitochondrial dysfunction. The proposed strategies 1–4 exhibit CISD2-preserving effects, which contribute to the prevention of inflammation and mitochondrial dysfunction. CISD2 augmentation can help overcome the detrimental effects of NFκB signaling and can be considered a potential therapeutic target for CNS injury or disease. In summary, novel strategies involving biomedical engineering or pharmacological novel agents that help CISD2 elevation can be suggested for the management of NDs.

## 9. Conclusions

**Figure 1:**
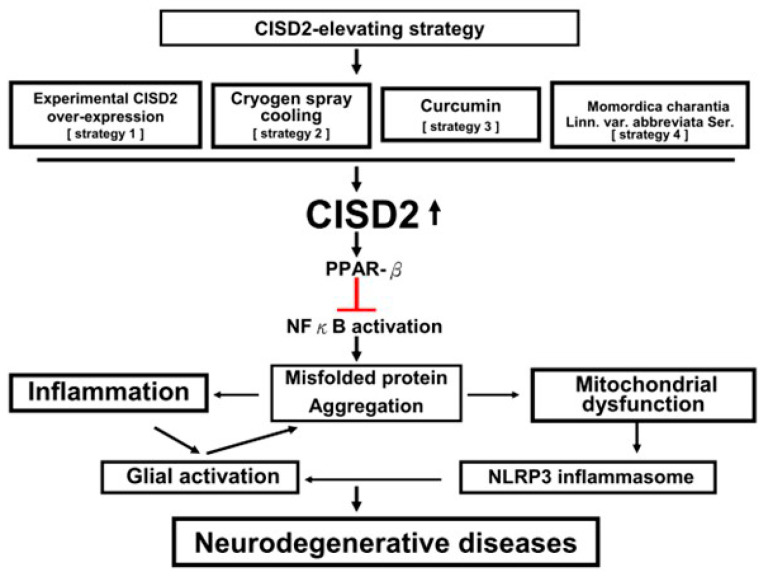
Diagram of the benefit of CISD2-elevating strategies in neurodegenerative diseases (NDs). NFκB-provoked inflammation and mitochondrial dysfunction underlie the pathogenesis of NDs. Accumulation of misfolded proteins in the central nervous system (CNS) induces neuroinflammation by triggering the activation of microglia, astrocytes, and glia-secreted proinflammatory cytokines/chemokines. Advanced neuroinflammation consequently leads to mitochondrial dysfunction and the production of mitochondrial damage-associated molecular patterns (DAMP), which promote the activation of NLRP3 inflammasome and subsequently exaggerate neuroinflammation. Through association with PPAR-β and prevention of IκB degradation, CISD2 can inhibit NFκB activation (red bar). Therapeutic strategies that upregulate CISD2 have the potential to attenuate the PPAR-β/IκB/NFκB signaling pathway and mitigate NFκB-provoked inflammation and mitochondrial dysfunction implicated in NDs.

With the increase in the aging population worldwide, the number of people affected by NDs may rise rapidly. This significant issue needs to be addressed. NDs commonly cause physical impairment and functional disability among the elderly population worldwide, increasing the burden on society. Several factors susceptible to this disease category include genetics, aging, and the environment, which may lead to abnormal aggregation of misfolded proteins in the CNS (Figure 1) and further trigger neuroinflammation, mitochondrial dysfunction, neuronal loss, and eventual neurological deficits.

As shown in this review article, the function of CISD2 is not limited to calcium metabolism, anti-apoptosis, and longevity. CISD2 functions as a family of NEET proteins along with zinc finger proteins possessing the [2Fe-2S] cluster. These properties enable the protein to regulate vital proteins, such as Bcl2, PPAR-β, NFκB, and CISD1. Through cluster reception/donation, CISD2 combined with Bcl2 and CISD1 can regulate the key physiological mechanisms of apoptosis-related mitochondrial function/mitochondrial inflammasome activation and mitochondrial Fe/ROS/Fe-S homeostasis, respectively. Upstream modulation of PPAR-β/NFκB proinflammatory signaling of CISD2 provides a rationale for the management of the two NFκB-provoked pathological mechanisms (neuroinflammation and mitochondrial dysfunction), which underlie NDs (Figure 1). Novel strategies targeted to elevate CISD2 are expected to be used in the management of NDs.
